# Genetic susceptibility to oral and atherosclerotic cardiovascular diseases based on dental and heart SCORE studies

**DOI:** 10.1038/s41598-025-18651-1

**Published:** 2025-09-26

**Authors:** Mariana Bezamat, Dylan J. Baxter, Nandita Mukhopadhyay, Anum Saeed, Lei Liu, Lisa de las Fuentes, Betsy Foxman, John R. Shaffer, Daniel W. McNeil, Steven E. Reis, Mary L. Marazita

**Affiliations:** 1 Department of Oral and Craniofacial Sciences, School of Dental Medicine, University of Pittsburgh, Pittsburgh, PA USA; 2 Heart and Vascular Institute, University of Pittsburgh Medical Center, Pittsburgh, PA USA; 3 Center for Biostatistics and Data Science, Institute for Informatics, Data Science, and Biostatistics (I2BD), School of Medicine, Washington University, St. Louis, MO USA; 4 Department of Medicine, Cardiovascular Division, School of Medicine, Washington University, St. Louis, MO USA; 5 Department of Epidemiology, University of Michigan School of Public Health, Ann Arbor, MI USA; 6 Department of Human Genetics, School of Public Health, University of Pittsburgh, Pittsburgh, PA USA; 7 Department of Community Dentistry and Behavioral Science, College of Dentistry, University of Florida, Gainesville, FL USA; 8 Center for Craniofacial and Dental Genetics, Department of Oral and Craniofacial Sciences, School of Dental Medicine, University of Pittsburgh, Pittsburgh, PA USA; 9 Clinical and Translational Science Institute, University of Pittsburgh, Pittsburgh, PA USA

**Keywords:** Genetic association study, Genomics, Population genetics, Biomarkers, Cardiology

## Abstract

Periodontal disease and dental caries are two oral conditions that have been associated with atherosclerotic cardiovascular disease (ASCVD). However, it is unclear if one of the key mechanisms involved in this association could be a shared genetic susceptibility. The goal of this study was to explore whether there is an intersection of genetic loci among individuals with comprehensive oral examinations and subclinical ASCVD screenings. We leveraged data from oral and medical examinations obtained from the Dental and Heart Strategies Concentrating on Risk Evaluation (Dental/Heart SCORE) projects. Genome-wide association studies (GWASs) were performed independently in 552 participants (aged 45–75 years). The decayed, missing, or filled teeth index (DMFT) and periodontal disease indices were used to reflect oral conditions; coronary artery calcium scores (CAC) and carotid intima media thickness (CIMT) were analyzed as subclinical ASCVD traits. Single nucleotide variant (SNV) associations with oral and ASCVD traits were found; however, there were only a few regions of suggestive genetic loci overlap between these conditions. The most robust associations found for each phenotype are as follows: DMFT with rs79198416 (near *CDC73/KCNT2*; *p* = 7.57E-07), periodontal disease with rs73870587 (*DIPK2A*, *p* = 7.38E-08); CIMT with rs113152669 (*LRP1B* *p* = 4.07E-07), and CAC with rs76676138 (*CNTNAP2*; *p* = 2.47E-19). Although genetic associations were identified for each of the phenotypes of interest in the GWASs, there were no regions of shared genetic loci that significantly intersected across phenotypes. Thus, our results suggest that incorporation of environmental, behavioral, microbiome-related factors, and larger sample sizes, are warranted in future studies between oral and cardiovascular health.

**Supplementary Information:**

The online version contains supplementary material available at 10.1038/s41598-025-18651-1.

## Introduction

Despite being highly preventable^1^dental caries is the most prevalent chronic disease in both children and adults^2^ with more than 1 in 5 people having untreated dental caries in the US population^3^. Different dental diseases have been suggested to affect the risk for systemic inflammatory conditions^1^, including atherosclerotic cardiovascular disease (ASCVD), the leading cause of death worldwide^4^. Subclinical ASCVD markers, such as the carotid intima-media thickness (CIMT) and coronary artery calcification (CAC), along with risk factors such as inflammatory dysregulation and lifestyle stressors, can predict future risk of cardiovascular outcomes^5^.

A suggested mechanism that may play a role in the correlation between oral disease and ASCVD is genetic susceptibility, determined in part by single nucleotide variants (SNVs) in or regulating genes involved in inflammatory pathways^6^. This was mainly suggested for the association between periodontal disease and ASCVD, with the association with dental caries being less explored. However, early life oral disease (mainly dental caries) was associated with higher burden of atherosclerotic cardiovascular disease in adulthood^7^. Thus, it is plausible that there is an interplay between dental caries and future risk of ASCVD, which usually begins decades before ASCVD manifests clinically.

Few studies have focused on identifying the overlap in genetic loci associated with dental caries and subclinical ASCVD^8^, and further research is needed to explore pleiotropic effects between periodontal disease and ASCVD. It is not clear whether periodontal disease and atherosclerosis are outcomes of similar maladaptive inflammatory pathways^6^. If this is the case, pleiotropic genes may affect the development of both periodontal disease and subclinical ASCVD^9^. Prior studies have suggested that the association between cardiovascular disease and periodontal disease may have been confounded by genetics^6^. However, to date, candidate gene association studies have only identified 5 genes that have been associated with both cardiovascular disease and periodontal disease traits (*PLG*,* CAMTA1*,* VAMP3*,* VAMP8 and CDKN2B*)^9–11^.

Addressing gaps in knowledge by determining whether pleiotropy between oral disease and subclinical ASCVD in fact exists will offer mechanistic insights into disease initiation and progression. We performed independent GWASs to explore potential pleiotropic regions using the combined Dental and Heart SCORE cohorts of 552 subjects. We hypothesize that there will be an intersection of genetic loci associated with oral conditions (i.e., dental caries and periodontal disease) and subclinical ASCVD (i.e., CIMT and CAC). In summary, establishing genetic relationships between these conditions may advance mechanistic understanding in disease susceptibility.

## Results

Table 1 presents the study participant demographics and cohort characteristics. Of the 552 subjects, CIMT measurements were obtained for 337 individuals, while 221 subjects had CAC assessment. Notably, data on both measurements were available for 145 participants. In total, 242 participants had periodontitis (44%), and the DMFT mean value was 16 in this cohort (ranging from 0 to 28).

**Table 1:** Participant demographics.

Age in years (mean, range)	63.1	(47–79)
Sex (*n*, %)		
Female	371	(67.2%)
Male	181	(32.8%)
Self-reported		
Race (n, %)		
Non-Hispanic White	298	(54%)
Non-Hispanic Black	226	(41%)
Asian	9	(1.6%)
Native American	2	(0.4%)
Pacific Islander	1	(0.2%)
More than one category/other/not reported	16	(2.9%)
Oral diseases		
Periodontitis (n, %)	242	(44%)
DMFT (mean, range)	16	(0–28)
Subclinical ASCVD (mean, range)		
CIMT, mm	0.8	(0.5–1.8)
CAC, Agaston units	117	(0-2081)
Smokers (n, %)	45	(8%)
Diabetes (n, %)	33	(6%)
Hypertension, Stage 1 or 2 (n, %)	240	(43.5%)
Total cholesterol, mg/dL (mean, range)	204	(103–349)

**Fig. 1:**
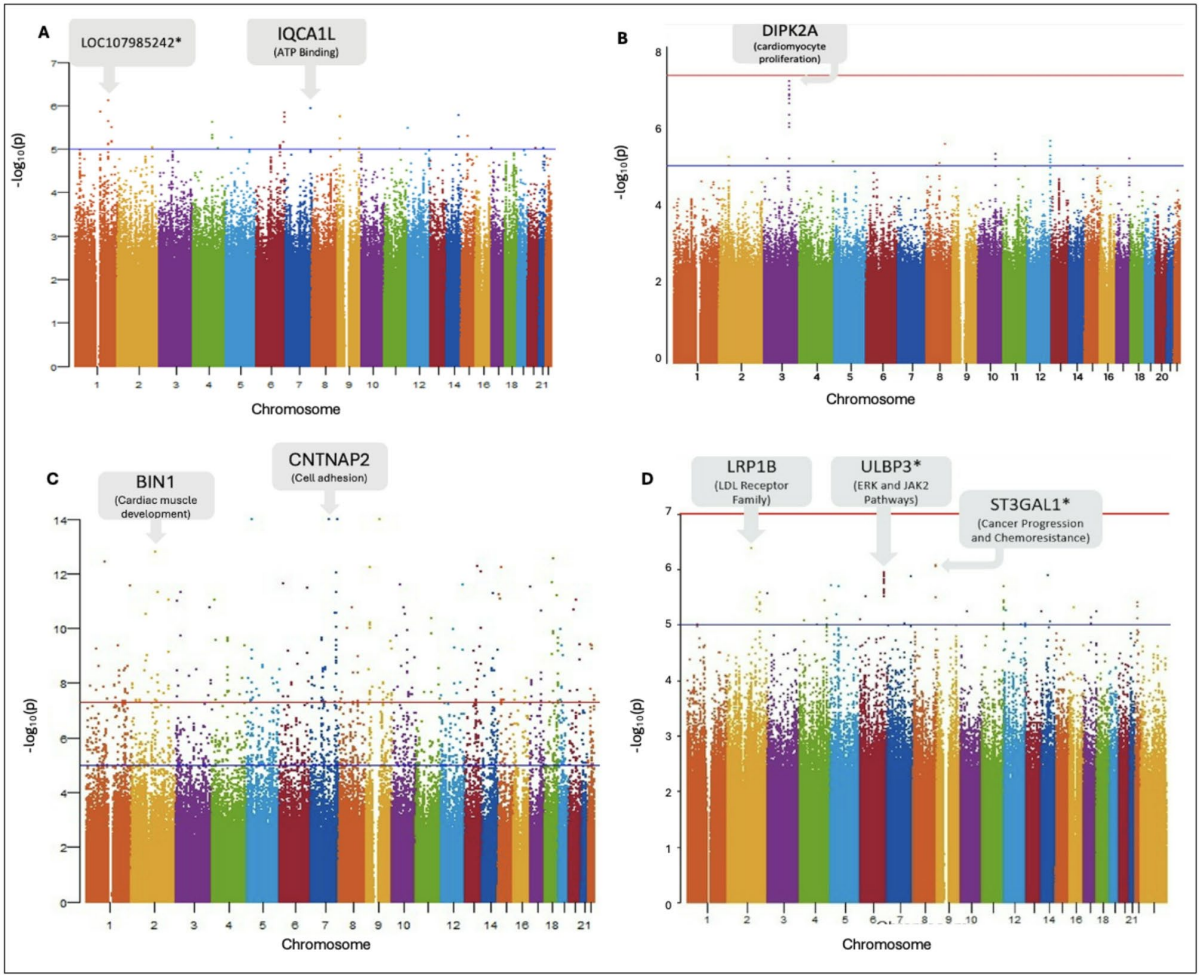
Manhattan plots showing all sequenced SNVs from individuals in the study for (**A**) DMFT, (**B**) PSR, (**C**) CAC, and (**D**) CIMT. Each dot represents the aggregate significance of an individual SNV. The blue line threshold represents suggestive associations (5 × 10^− 5^), and the red line threshold represents significant associations (5 × 10^− 8^). There are approximately 8.5 million SNVs represented and the most significant SNVs associated with each GWAS trait are annotated. Genes marked with an asterisk (*) indicate that the SNV is located near, but not within, the annotated gene.

The Manhattan plots show the SNVs most strongly associated with each phenotype (Fig. 1A–D). Specific associations for each of the top hits are provided in Supplementary Tables 1–4 and Q-Q plots in Suplemmentary Figs. 3, 4, 5 and 6. Genome-wide significant associations were only identified for the CAC phenotype (Fig. 1C, Supplementary Table 3).

The GWAS results for quantitative DMFT scores found suggestive associations with several SNVs (Fig. 1A, supplementary Table 1). Although these results did not reach genome-wide significance of 5 × 10^− 8^, interesting suggestive associations were identified for the rs79198416 (LOC107985242, near *CDC73/KCNT2)*; *p* = 7.57 × 10^− 7^ and for the rs116679466 in *IQCA1L*, *p* = 1.15 × 10^− 6^.

For PSR, there were suggestive associations noted; the rs16854752 (*p* = 7.44 × 10^− 8^) is located in the *divergent protein kinase domain 2 A (DIPK2A)* gene and several other suggestive SNVs are in chromosome 3 (Fig. 1B, supplementary Table 2).

The CAC phenotype showed associations with several SNVs, including rs76676138, rs375047716, rs34729913, rs112265837, rs148981230 (Fig. 1C, supplementary Table 3). The strongest signal was found for SNV rs76676138 which is located near the *CCNB3P1* pseudo gene. The other SNVs are located within genes. SNV rs375047716 is in *CNTNAP2*, and rs34729913 in the uncharacterized area LOC101927281; rs112265837 located in *ORAI2*. SNV rs148981230 is located in *BIN1.*

The CIMT phenotype was associated with different SNVs including the rs113152669 in *LRBP1*; and rs72722175 near *ULBP3* (Fig. 1D, supplementary Table 4).

**Table 2:** Top CAC significant associations intersecting with DMFT and PSR suggestive associations.

CAC significantly associated locus	rs number (bp position) of CAC lead variant	*p*-value of lead CAC variant	Strongest association seen near CAC lead variant for DMFT		Strongest association seen near CAC lead variant for PSR	
			*p*-value	rs number (bp position)	*p*-value	rs number (bp position)
17p13.3	rs139699557 (1,393,514)	3.05E-12	9.38E-06	rs9330548 (1,134,457)	-	-
3p25.3	rs142489717 (8,484,161)	1.03E-11	-	-	3.44E-05	rs712788 (7,487,990)
3p24.2	rs115488988 (24,943,736)	4.60E-12	-	-	6.49E-06	rs826217 (24,269,789)
11q14.2	rs74393381 (86,891,074)	1.60E-10	9.81E-06	rs2512314 (88,205,762)	2.23E-05	rs59016931 (85,057,399)
			9.81E-06	rs10765563 (88,217,400)		
2q33.1	rs79523653 (202,743,211)	9.33E-12	1.52E-05	rs16838147 (202,645,961)	-	-
1q43	rs72761235 (242,292,022)	2.72E-12	6.90E-06	rs320467 (213,652,148)	3.02E-05	rs12735074 (214,448,561)
			3.14E-06	rs320470 (213,657,774)	2.60E-05	rs6691532 (214,466,553)
7q35	rs375047716 (146,682,394)	2.47E-19	7.57E-07	rs116679466 (150,888,426), 7q36.1	-	-
	rs11767967 (144,957,045)	7.6E-12			-	-

Lastly, there were no regions of shared genetic loci that intersected significantly across phenotypes when a genetic distance of a maximum of 1 Mb was considered for shared genetic associations between traits (*p* < 5 × 10^− 8^). The closest significant overlap was identified between the rs375047716 and rs11767967 with CAC levels and the rs116679466 with higher DMFT scores (*p* = 7.57 × 10^− 07^, Table 2). The SNV rs375047716 is located in chromosome 7, *CNTNAP2* gene. It is approximately 4 million bp away from rs116679466. None of these SNVs are in linkage disequilibrium (Supplementary Fig. 2). However, there were several genetic loci that overlapped between significant CAC results (*p* < 5 × 10^− 8^) with suggestive (*p* < 5 × 10^− 5^) DMFT and PSR results (Table 2).

## Discussion

Assessments of genetic loci associated with both oral disease and ASCVD reported four genetic loci associated with both traits (reviewed by Loos^6^. Additionally, *PLG* variants have been reported as associated with both chronic and aggressive periodontitis^12^ and this gene has been suggested to also play a role in atherosclerosis in clinical studies^13,14^. Nevertheless, most of these studies were conducted using separate datasets for oral health and ASCVD^10,15^. In contrast, the present study utilizes a cohort with both oral and ASCVD data collected from the same individuals.

There were no SNVs that significantly intersected across the phenotypes. However, there were several associations with each phenotype individually that are of note. For the DMFT phenotype, SNVs rs79198416 and rs116679466 showed suggestive associations approaching genome-wide significance. The rs79198416 variant lies within an uncharacterized locus LOC107985242 which requires further investigation. The nearest gene is the *EEF1A1P14* which is in a genetic locus that has been associated with childhood obesity in a Hispanic population^16^. The rs116679466 is located within the *IQCA1L* gene which has been associated with ATP binding^17^. The PSR phenotype had several SNVs located in the *DIPK2A* gene that has been shown to have expression in cardiomyocyte proliferation^18^, which is a novel finding. *DIPK2A* is involved with other processes including negative regulation of smooth muscle cell apoptotic process, positive regulation of protein kinase C activity, and autophagy^19^. It has also been demonstrated to inhibit apoptosis and enhance cell survival^19^. Interestingly, the products of *DIPK2A* and *VAMP8* have been shown to interact^19^ which is a gene previously identified to overlap in coronary artery disease and periodontitis^15^. The CAC phenotype showed significant association with SNVs rs76676138, rs373047716, rs34729913, rs112265837, and rs148981230, to name a few. SNV rs76676138 is located near the *CCNB3P1* pseudogene. SNV rs373047716 is located in the *CNTNAP2* gene which is the largest gene on chromosome 7 and has been associated with multiple neurodevelopmental disorders^20^. BIN1 is highly relevant in age-related cognitive decline^21^ but the identified SNV (rs148981230) has never been specifically reported as associated with disease in the literature. The SNV rs112265837 is located in the *ORAI2* gene which has been previously associated with calcium channel activity in the plasma membrane and oral cancer cell migration^22^. The CIMT phenotype was associated with rs113152669 in *LRP1B* gene which belongs to the LDL receptor family, and several SNVs in or near *ULBP3* gene.

Previously identified associations between dental caries and ASCVD may have been confounded by diet and the microbiome, while an involvement of a common SNV seems unlikely in this population according to our results. Dental caries occurs when oral bacteria, especially *Streptococcus mutans*, metabolize sugars, producing acids that demineralize enamel^23,24^. This bacterium is not only a key factor for caries but has also been found in cardiovascular specimens, such as heart valves and atheromatous plaques^25^.

On the other hand, periodontal disease is characterized by inflammation of the gingival tissue and the bone that supports the teeth. This chronic, low-grade inflammation is thought to contribute to a systemic inflammatory response. The inflammation is triggered when bacteria infiltrate periodontal pockets, prompting an immune response from the host. Without appropriate treatment, such bacterial infections can lead to the loss of alveolar bone, loss of teeth, and other complications related to oral health. The two main proposed mechanisms involved with the connection between periodontal disease and ASCVD suggest that subgingival pathogens may directly invade endothelial cells, or an indirect mechanism created by elevated levels of inflammatory cytokines due to periodontitis stimulating a chronic inflammatory response. The first mechanism is supported by the presence of periodontal disease bacteria formed in atherosclerotic lesions in the coronary arteries and the second by the presence of increased levels of IL-1b, IL-6, IL-8 and TNF-a which are cytokines also related to ASCVD^26^. Interestingly, we recently identified a shared sphingomyelin metabolite in gingivitis and CIMT in this same cohort, which may explain some of these mechanisms^27^.

In summary, we did not identify significantly associated genetic loci contributing to both oral and atherosclerotic cardiovascular diseases. We found some suggestive intersections that included 1q43, 2q33.1, 3p24.2, 3p25.3, 11q14.2, and 17p13.3 loci that may require further investigation using larger sample sizes. The lack of significant results indicates that the associations between oral health and ASCVD observed in previous studies may be less attributable to shared genetic susceptibility and more influenced by environmental, behavioral, and microbiome-related factors, potentially pointing to a more direct causal relationship. However, this present exploratory study had noted limitations. Given the cross-sectional design, we cannot determine causality between oral conditions and ASCVD to confirm a causal relationship. Additionally, as mentioned before, our sample size could have been a limiting factor in detecting significant associations and a potential overlap. We have also established a liberal significance threshold of 5 × 10^− 8^ in our GWASs analyses which may have led to finding potential false positive results for CAC trait. Nevertheless, strengths of the study include the presence of precise oral and cardiovascular phenotypes in the Dental/Heart SCORE cohort and the presence of whole genome sequencing data that allowed us to conduct comprehensive GWASs in this population. In conclusion, future studies should explore additional shared multi-omics influences to elucidate mechanistic insights affecting oral and atherosclerotic cardiovascular diseases.

## Methods

### Participants

The research participants were recruited through a community-based study called Heart Strategies Concentrated on Risk Evaluation (Heart SCORE) that began in 2003 and enrolled 2,000 participants. The study’s primary goal was to investigate racial disparities in cardiovascular disease^28^. Participants were age 45 to 75, lived in the greater Pittsburgh metropolitan area, and free of known health conditions likely to restrict life expectancy to less than five years. Dental assessments and related data collection were carried out between 2007 and 2010 on a subgroup of 552 participants, known as the Dental Strategies Concentrating on Risk Evaluation (Dental SCORE) study.

All participants in the Heart/Dental SCORE studies provided written informed consent, as approved by the University of Pittsburgh Institutional Review Board. The procedures strictly adhere to regulations and protocols set forth by the Center for Oral Health Research in Appalachia, cohort 1 (COHRA1). The COHRA1/Dental SCORE protocols and genetic data are accessible on dbGaP (accession number phs001591.v1. p1), and extended phenotypic data are available on FaceBase (FaceBase.org, DentalSCORE Record ID: 3P4VSA, Accession: FB00001361, DOI: 10.25550/3P-4VSA).

The cohort data contain comprehensive information on subclinical markers of ASCVD, clinical variables such as blood pressure (i.e., normal, prehypertensive, hypertensive stage 1, and hypertensive stage 2), diabetes, smoking status, HDL cholesterol, and total cholesterol, and self-reported demographic details (including race and ethnicity) for all participants^28,29^. Participants were categorized based on their smoking habits as current, former, or never smoker.

### Phenotypes assessed

#### Oral health status phenotypes

Oral examinations were carried out by experienced dental professionals, including dentists, dental hygienists, and dental assistants. Inter- and intra-examiner concordances were high as reported elsewhere^29,30^. The assessment included (i) dental caries, assessed using the decayed, missing (due to caries), and filled teeth index (DMFT) as a continuous trait, and (ii) periodontal health, evaluated with the Periodontal Scoring and Recording index (PSR). For the PSR trait, we assigned a pocket depth score to each sextant of an individual’s mouth based on the most severe finding. Scores were 1 (less than 3.5 mm of pocket depth), 2 (3.5 to 5.5 mm of pocket depth), and 3 (more than 5.5 mm of pocket depth). We used the highest PSR score among the sextants (numbered 1–3) in the analysis.

#### ASCVD phenotypes

Two subclinical markers of ASCVD were assessed in this study, carotid artery intima-media thickness (CIMT) and coronary artery calcification (CAC). CIMT was measured in millimeters using ultrasound and codified as a continuous trait with the highest value between right and left being used for analysis. Electron-beam computed tomography (EBCT) was used to assess CAC (total volume in millimeters) values and measure the severity of calcification^31^ as a continuous trait. The CAC score was determined using the Agatston score, which is the sum of the attenuation (in Hounsfield units) and area of CAC lesions in the coronary arteries^32^.

### Genome-wide association analyses and intersection between traits

GWASs were carried out separately on each of the four phenotypes. Study participants were genotyped on the Illumina Infinium Multi-Ethnic Global-8 v1.0 array and subsequently imputed using the Michigan Imputation Server based on the HRC (Version r1.1 2016) reference panel, genome build GRCh37/hg19. Approximately 8.5 million SNVs that met Hardy Weinberg equilibrium (HWE), and imputation quality filtering thresholds^33^ were initially considered for the GWASs, and further filtered for minor allele frequency as appropriate based on the sample size available for each GWAS. Sex and age were included as covariates in the regression in each GWAS as well as principal components of ancestry to account for population admixture.

Genome wide p-value thresholds of 5 × 10^− 8^ and 10^− 5^ were used to identify significant and suggestive associations respectively. SNVs were annotated to genes including or adjacent to the SNV. We also examined whether the associated loci from any of our GWASs overlapped between the four (oral and ASCVD) phenotypes. For the purpose of this comparison, search intervals of 1 megabase length were drawn around suggestive and significant loci from each GWAS and p-values were examined across all four GWASs within each search interval.

The analysis was completed using PLINK genetic analysis software (version 2.050)^34^ including the 3 most significant principal components of ancestry to adjust for population substructure (Supplementary Fig. 1). Linear regression was used to analyze DMFT, CIMT, and CAC; and PSR was analyzed using a logistic regression framework. The Fastman R package^35^ was used to generate Manhattan and Q-Q plots.

## Supplementary Information

Below is the link to the electronic supplementary material.

Supplementary Material 1

## Data Availability

The dataset generated and/or analyzed during the current study are available from the corresponding author on reasonable request. The COHRA1/DentalSCORE protocols and genetic data are accessible on dbGaP (https://www.ncbi.nlm.nih.gov/gap; accession number phs001591.v1. p1), and extended phenotypic data are available on FaceBase (FaceBase.org; DentalSCORE Record ID:3P4VSA Accession: FB00001361 DOI:10.25550/3P-4VSA).
