# Supplementary material for: Genetic susceptibility to oral and atherosclerotic cardiovascular diseases based on dental and heart SCORE studies
Source: Sci Rep. 2025 Sep 26;15:33257. doi: 10.1038/s41598-025-18651-1 (PMC12475168; doi:10.1038/s41598-025-18651-1)
Supplement: Supplementary file 1 — Supplementary Material 1 [file 41598_2025_18651_MOESM1_ESM.pdf]

Supplementary material:

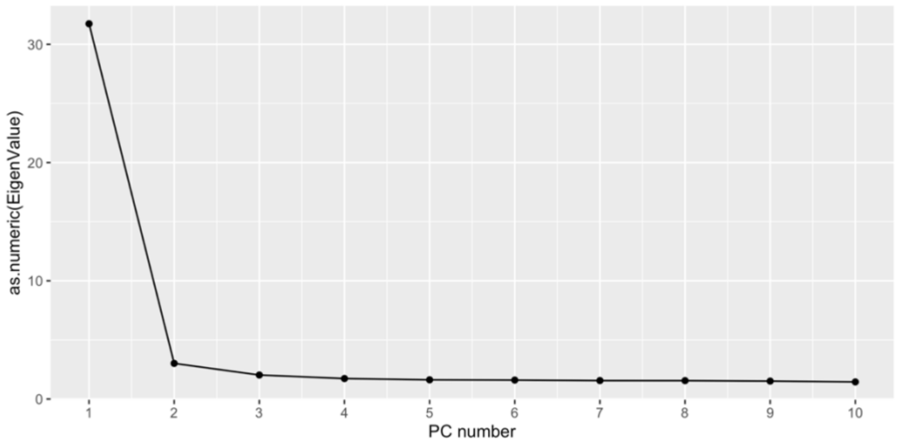

Supplementary Figure 1. Scree plot of Principal Component Analysis. The plateauing of the eigenvalues after the principal component 3 can be visualized in the plot; thus, principal components 1-3 were used to control for population substructure in the subsequent GWASs.

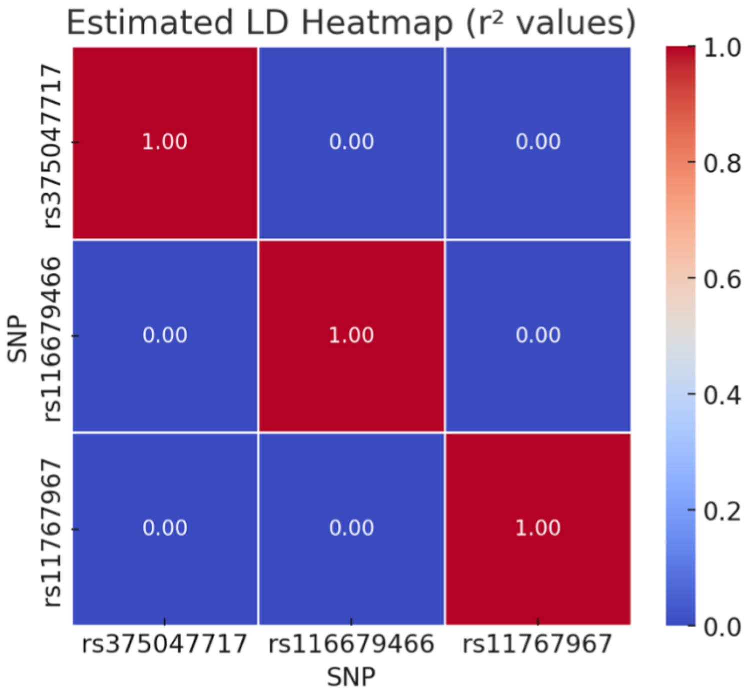

Supplementary Figure 2. Results of the linkage disequilibrium analysis of the three DMFT and CAC SNVs identified as being located approximately 2-4 million bp from each other.

Supplementary Table 1. Top 15 DMFT associated SNVs.

CHR	SNV	POSITION	P	Gene
1	rs79198416	194335553	7.57E-07	LOC107985242*
7	rs116679466	150888426	7.57E-07	IQCA1L: Intron Variant
1	rs2992456	147804327	1.15E-06	LOC124904404: 2KB Upstream Variant
6	rs12179628	169544139	1.39E-06	LINC01615*
6	rs9688472	169544750	1.44E-06	LINC01615*
14	rs11846389	93779117	1.47E-06	BTBD7: Intron Variant
9	rs57467578	15512821	1.69E-06	PSIP1: 2KB Upstream Variant
9	rs62571021	15513536	1.72E-06	PSIP1*
9	rs62571022	15513907	1.72E-06	PSIP1*
9	rs62571023	15514051	1.72E-06	PSIP1*
9	rs62571024	15514278	1.72E-06	PSIP1*
9	rs62571027	15515899	1.72E-06	PSIP1*
6	rs10945382	169543531	1.79E-06	LINC01615*
1	rs4609407	194335342	1.82E-06	LOC107985242*
4	rs7694909	111195127	2.31E-06	ZBED1P1*

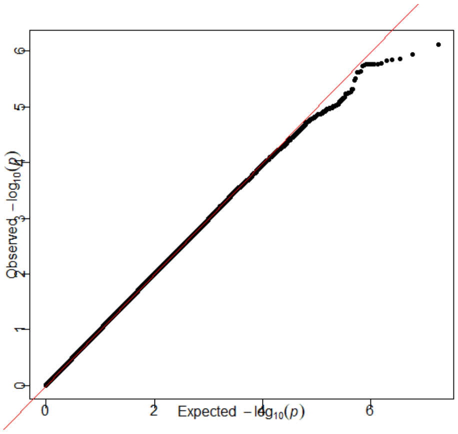

Supplementary Figure 3.DMFT Q-Q plot.

Supplementary Table 2. Top 15 PSR associated SNVs.

PSR	CHR	SNV	POSITION	P	Gene

3	rs73870587	143665939	7.38E-08	DIPK2A*
3	rs16854752	143689684	7.441E-08	DIPK2A: 2KB Upstream Variant
3	rs73870586	143665853	7.441E-08	DIPK2A*
3	rs73872667	143686488	7.441E-08	DIPK2A*
3	rs7643008	143669057	9.602E-08	DIPK2A*
3	rs73870585	143662764	1.183E-07	DIPK2A*
3	rs78983291	143643928	1.549E-07	DIPK2A*
3	rs7636333	143679448	1.686E-07	DIPK2A*
3	rs111932429	143650528	1.998E-07	DIPK2A*
3	rs73872624	143682596	2.624E-07	DIPK2A*
3	rs56805765	143704110	5.266E-07	DIPK2A: Intron Variant
3	rs28475588	143695952	8.617E-07	DIPK2A: Intron Variant
3	rs56367637	143699272	1.07E-06	DIPK2A: Intron Variant
3	rs57432713	143699273	1.07E-06	DIPK2A: Intron Variant
3	rs73870994	143700969	1.07E-06	DIPK2A: Intron Variant

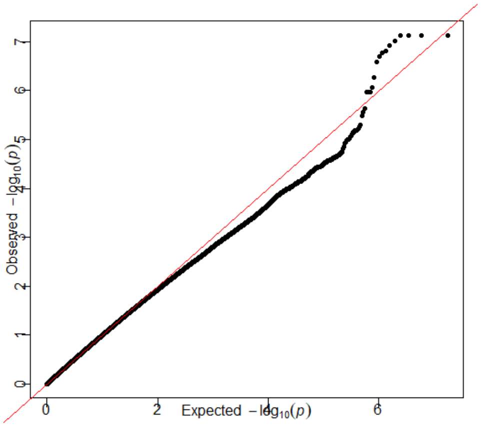

Appendix Figure 4. PSR Q-Q plot.

Supplementary Table 3. Top 15 CAC associated SNVs.

CHR	SNP	Position	P	Gene
5	rs76676138	26783209	1.03E-19	CCNB3P1*
7	rs375047716	146682394	2.47E-19	CNTNAP2: Intron Variant

9	rs34729913	75941235	7.68E-17	LOC101927281: Intron Variant
7	rs112265837	102091541	4.29E-16	ORAI2: 3 Prime UTR Variant
2	rs148981230	127839698	1.59E-13	BIN1: Intron Variant
18	rs111349964	46653245	2.84E-13	DYM: Intron Variant
18	rs113219267	46668256	2.84E-13	DYM: Intron Variant
1	rs72730349	99413510	3.86E-13	PLPPR5: Intron Variant
13	rs4351950	84615437	5.37E-13	LOC105370287*
9	rs76670734	23426931	5.71E-13	LOC105369298*
15	rs143510802	34765438	5.75E-13	HNRNPLP2*
13	rs71435301	109428831	8.11E-13	MYO16: Intron Variant
7	rs11767967	144654138	9.35E-13	EI24P4*
18	rs142219177	41224340	2.08E-12	LOC105372088*
6	rs4712499	20447293	2.39E-12	E2F3: Intron Variant

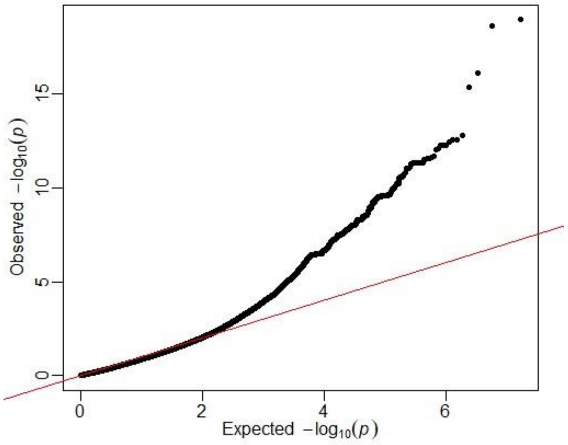

Supplementary Figure 5. CAC Q-Q plot.

Supplementary Table 4. Top 15 CIMT associated SNVs.

CHR	SNV	BP	p-value	Gene
2	rs113152669	142473137	4.07E-07	LRP1B: Intron Variant
8	rs72722175	134629687	8.36E-07	LINC03024*

8	rs72722176	134630941	8.85E-07	LINC03024*
6	rs11754434	150410985	1.13E-06	ULBP3*
6	rs9322242	150406035	1.21E-06	ULBP3*
6	rs9371701	150406604	1.21E-06	ULBP3*
6	rs789829	150408111	1.24E-06	ULBP3*
14	rs77212578	56760325	1.28E-06	PELI2: Intron Variant
6	rs789828	150408364	1.33E-06	ULBP3*
6	rs789827	150408440	1.33E-06	ULBP3*
6	rs789826	150408681	1.33E-06	ULBP3*
6	rs789825	150408946	1.33E-06	ULBP3*
6	rs789824	150409072	1.33E-06	ULBP3*
6	rs7747671	150409087	1.33E-06	ULBP3*
6	rs7755204	150409088	1.33E-06	ULBP3*

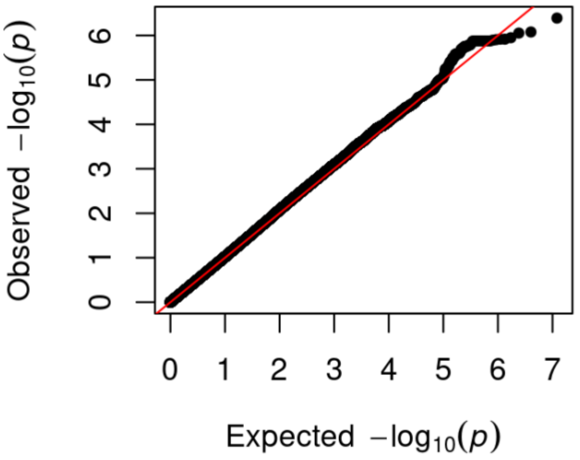

Supplementary Figure 6. CIMT Q-Q plot.
